# Individual Differences in Hippocampal Volume as a Function of BMI and Reward Sensitivity

**DOI:** 10.3389/fnbeh.2020.00053

**Published:** 2020-04-09

**Authors:** Maria Antònia Parcet, Jesús Adrián-Ventura, Víctor Costumero, César Ávila

**Affiliations:** ^1^Neuropsychology and Functional Neuroimaging, Jaume I University, Castellón, Spain; ^2^Center for Brain and Cognition, Pompeu Fabra University, Barcelona, Spain

**Keywords:** reward sensitivity, body mass index, hippocampus, voxel-based morphometry, obesity, reinforcement sensitivity theory

## Abstract

Sensitivity to reward is a personality trait that predisposes a person to several addictive behaviors, including the presence of different risky behaviors that facilitates uncontrolled eating. However, the multifactorial nature of obesity blurs a direct relationship between the two factors. Here, we studied the brain anatomic correlates of the interaction between reward sensitivity and body mass index (BMI) to investigate whether the coexistence of high BMI and high reward sensitivity structurally alters brain areas specifically involved in the regulation of eating behavior. To achieve this aim, we acquired T1-weighted images and measured reward sensitivity using the Sensitivity to Punishment and Sensitivity to Reward Questionnaire (SPSRQ) and BMI in a sample of 206 adults. Results showed that reward sensitivity and BMI were not significantly correlated. However, neuroimaging results confirmed a relationship between BMI and reduced volume in the medial and lateral orbitofrontal cortex, and between reward sensitivity and lower striatum volume. Importantly, the interaction between the two factors was significantly related to the right anterior hippocampus volume, showing that stronger reward sensitivity plus a higher BMI were associated with reduced hippocampal volume. The hippocampus is a brain structure involved in the higher-order regulation of feeding behavior. Thus, a dysfunctional hippocampus may contribute to maintaining a vicious cycle that predisposes people to obesity.

## Introduction

Feeding is a frequent behavior that is modulated by a plethora of external and internal factors (Berthoud, 2002). Typically, feeding behavior is a motivated behavior directed toward satisfying hunger as a basic physiological need. However, food stimuli are omnipresent in the western world, which turns feeding behavior into frequent alternations between binge-eating episodes and restrained eating behavior (Razzoli et al., 2017). In these situations that present approach-avoidance conflicts, diverse factors, including personality, may determine individual differences in sensitivity to food stimuli.

Obesity is currently considered a pandemic neurobehavioral disorder (Kopp, 2019). Risk factors can be categorized as domain-general (i.e., personality, motivation) and eating-specific (i.e., hunger, appetizing foods). The main general risk factor described is impulsivity, defined as a “tendency to pursue rewards without full consideration of the negative consequences of the actions” (Neseliler et al., 2018). According to the neuropsychological framework offered in the Reinforcement Sensitivity Theory (Gray and McNaughton, 2000), this kind of impulsivity is associated with the activity of the Behavioral Activation System (BAS). The BAS is a reward system rooted in the striatum and the dopaminergic pathways toward the prefrontal cortex and the limbic system. Individual differences in BAS activity determine a personality trait known as reward sensitivity that has been associated with a higher probability of detecting reward cues in the environment and emitting approach responses toward these stimuli (Avila et al., 2008). Neuroimaging studies have demonstrated that reward sensitivity was associated with reduced volume in the striatum and the prefrontal cortex (Barrós-Loscertales et al., 2006a; Holmes et al., 2016; Adrián-Ventura et al., 2019) and more activity in the same areas while processing reward cues (Carter et al., 2009; Hahn et al., 2009; Costumero et al., 2013). In the field of obesity, personality traits such as reward sensitivity have been related to the presence of different risky behaviors leading to obesity, such as overeating, food cravings, episodes of binge-eating, and a preference for unhealthy foods (Loxton and Dawe, 2001; Davis et al., 2004, 2007; Franken et al., 2006). Furthermore, an fMRI study showed that activity in reward-related brain areas, including the striatum, midbrain, and right orbitofrontal cortex, was higher in individuals with stronger reward sensitivity while watching pictures of appetizing foods (Beaver et al., 2006).

The evidence showing a direct relationship between reward sensitivity and obesity is mixed. Some correlational studies investigating the relationship between self-reported measures of reward sensitivity and BMI have confirmed a positive correlation (Franken and Muris, 2005; De Decker et al., 2016), or a quadratic relationship (Davis and Fox, 2008; Dietrich et al., 2014), but others have reported no significant correlation (Matton et al., 2013; Vandeweghe et al., 2017; Jonker et al., 2019). A second approach consisting of comparing obese and lean groups also demonstrated stronger reward sensitivity in the former group in some studies (van den Berg et al., 2011), but no significant between-group differences in others (Nederkoorn et al., 2006; Schienle et al., 2009). Thus, although there is some evidence supporting the positive association of the role of reward sensitivity, many other factors may influence the development of obesity.

Importantly, the RST model also predicts that the BAS influences the Behavioral Inhibition System (BIS), located in the septohippocampal area when coping with approach-avoidance conflicts (Gray and McNaughton, 2000). In other words, when the presence of a reward cue is accompanied by the possibility of negative consequences (for instance, the presence of appetizing and unhealthy food), the activity of the reward system is also modulated by the limbic system (the BIS). Evidence in humans has shown the important role of the hippocampus in these conflicting situations (Loh et al., 2017). Behavioral evidence shows that individuals with stronger reward sensitivity have a greater probability of perseverating in the previously rewarded behavior, despite the possibility of punishment (Avila, 2001; Ávila and Torrubia, 2004). The proposed mechanism in this impulsive behavior is a dysfunctional BIS that minimizes reflectivity about the negative consequences of the behavior (Patterson and Newman, 1993; Avila et al., 2008). In the specific context of food-related behavior, several recent proposals have described the hippocampus as the key brain area involved in integrating episodic memories, the external context, and interoceptive signals, to regulate feeding behavior (Hargrave et al., 2016; Kanoski and Grill, 2017; Stevenson and Francis, 2017). Specifically, these proposals suggest that a hippocampal dysfunction may promote overeating by weakening the ability of satiety cues to withdraw food-related behavior and increasing the probability of engaging in unhealthy behaviors. Despite these proposals, the precise link between the reward system, the hippocampus, and the overweight has not been demonstrated. A recent study established this relationship in adolescents, showing that both impulsivity and BMI were correlated positively with stronger functional connectivity at rest between the striatum and the hippocampus (Sharkey et al., 2019).

One of the most relevant areas of obesity research has focused on the neuroanatomic correlates. The global pattern is that BMI is negatively associated with overall gray volume at all ages (Hamer and Batty, 2019). However, the different morphometric studies have revealed two main patterns of results as a function of age (Carnell et al., 2012). Some studies have associated overweight and obesity with changes in cerebral morphology and functional competence in a similar way to pathological aging. There is growing evidence that obesity is an additional risk factor for cognitive impairment and dementia, among others, throughout the adult lifespan (Jagust et al., 2005). Different studies investigating anatomic correlates of BMI in middle-aged and older populations have reported a pattern of correlates focused on general brain atrophy, with special emphasis on the hippocampus (Raji et al., 2010). The second pattern of results has mainly been found in morphometric studies in younger populations. In these studies, BMI was negatively related to the gray matter (GM) volume in the right lateral and medial orbitofrontal cortex (Medic et al., 2016; Vainik et al., 2018). Neuropsychological studies with brain lesioned patients have reported that lesions in the ventromedial prefrontal cortex lead to deficits in the integration of information (Pelletier and Fellows, 2019) and that lesions in the right ventrolateral cortex lead to problems in inhibitory control (Aron et al., 2004). Thus, these BMI results reinforce the idea of deficits in executive functions and inhibitory control as mechanisms leading to overweight at an early age (Alonso-Alonso and Pascual-Leone, 2007).

The current study aimed to investigate the relationship between BMI, Sensitivity to Reward (SR), and brain volume in some regions that are part of the reward system in a sample of university students. Our fundamental interest was to demonstrate the effect of the interaction between BMI and SR on GM volume. This analysis will provide information about the brain effects of participants with a high BMI associated with high reward sensitivity. Our hypotheses are: (1) BMI anatomic correlates in our sample will be observed in lower OFC volumes; (2) SR correlates will be observed in reduced striatum volume, and (3) the interaction between high reward sensitivity and high BMI will be directly associated with the decrease in the volume of the hippocampus.

## Materials and Methods

### Participants

Two-hundred and six participants (107 males, 99 females; mean age = 23.65, *SD* = 6.67; range = 18–49 years) who participated in different fMRI studies were included in this research after signing an informed consent approved by the Jaume I University. All the participants were recruited from the same geographical area through email advertisements and word of mouth. Most of the participants were undergraduates (91.7%). Participants were interviewed by phone to rule out a history of neurological or psychiatric disorder or any other major medical problem. The experiment was approved by the Ethical Committee of the University Jaume I (Spain).

### BMI and Sensitivity to Reward

Height and weight were measured before scanning. We calculated the BMI of each participant, based on weight and size, using the following formula: BMI = weight/size^2^, and expressed in kg/m^2^. Immediately before the scanning, all the subjects completed the Sensitivity to Punishment and Sensitivity to Reward Questionnaire (SPSRQ; Torrubia et al., 2001). This personality questionnaire included the sensitivity to reward (SR) scale, which measures individual differences in the functioning of the reward system. This personality trait is associated with individual differences in the activity of the Behavioral Approach System (BAS), such that a high SR score facilitates appetitive learning and promotes disinhibited responses when responding to rewards and is related to addiction (Torrubia et al., 2008). The scale is composed of 24 yes/no items that measure individual differences in sensitivity to various rewards, including reinforcers such as money, sexual partners, social recognition, power, or loss of sensations, which describe heterogeneous situations in promoting responses to obtain rewards. Some representative scale items are: “Do you generally give preference to activities that involve an immediate gain?” or “Would you like to be a socially powerful person?” The SR score was obtained by adding the yes responses.

### MRI Acquisition and Voxel-Based Morphometry

All participants underwent an MRI scan using a 1.5-T Siemens Avanto scanner (Erlangen, Germany). We acquired a 3D MPRAGE T1 sequence (TE = 3.8 ms; TR = 2,200 ms; flip angle = 15°; matrix = 256 × 256 × 160 mm; voxel size = 1 mm^3^), and we applied the voxel-based morphometry toolbox to analyze the data (VBM8 version r445^1^). These preprocessing steps were followed: segmentation of the images into gray matter, white matter, and cerebrospinal fluid; affine registration to a standard ICBM template; DARTEL normalization of the GM segments to the MNI template; and modulation by nonlinear components derived from spatial normalization. A data quality check was made after preprocessing by analyzing sample homogeneity using covariance. We did not identify any statistical outliers. Finally, an 8-mm FWHM Gaussian kernel was applied to spatially smooth the images.

### Statistical Analysis

Whole-brain voxel-wise regression analyses were performed within the framework of the general linear model (GLM) in SPM8 (Friston et al., 1994). Three separate GLM analyses were performed: (1) To replicate previous findings relating brain volume and BMI, we carried out a regression analysis including GM volume as the dependent variable, BMI as the independent variable of interest, and age and sex as the independent variables of no interest (equation Y = β_BMI_ × BMI); (2) To replicate previous investigations relating brain volume and SR, we performed a regression analysis including GM volume as the dependent variable, SR as the independent variable of interest, and age and sex as the independent variables of no interest (equation Y = β_SR_ × SR); and (3) to test a possible interaction effect between BMI and SR on brain volume, a regression analysis was conducted taking GM volume as the dependent variable and the interaction between BMI and SR (BMI × SR) as the independent variable of interest, whereas age, sex, BMI, and SR were included as covariates of no interest (equation Y = β_BMI_ × BMI + β_SR_ × SR+ β_BMI/sr_ × BMI × SR). Finally, we applied an absolute threshold masking of 0.10 and a non-stationary smoothing correction in all the analyses.

For the whole-brain analyses, the statistical criterion was set at *p* < 0.05, FWE cluster-corrected for multiple comparisons with an auxiliary uncorrected threshold of *p* < 0.001. We also investigated volumetric differences in subcortical* a priori* regions of interest (ROIs). Based on our hypotheses, the nucleus accumbens and caudate nuclei were selected as ROIs for the SR analyses because both structures are key areas of the reward system.

*A priori* ROIs (left and right NAcc, caudate and hippocampus) were defined for each hemisphere using the probabilistic Neuromorphometrics atlas^2^. We employed a MATLAB script^3^ to obtain the modulated GM volumes (without smoothing) for each ROI. Then, we calculated partial correlations (one-tailed based on* a priori* hypotheses) using SPSS (v25) with these ROI GM volumes, SR scores, and the BMI × SR interaction as variables, and age and sex as covariates. Because we used 4 ROIs for the analyses (i.e., the striatum ROIs), the statistical threshold for multiple comparisons (*p* < 0.05 FWE) was set at *p* < 0.0125, whereas for the BMI × SR interaction, it was set at *p* < 0.025 (two ROIs; left and right hippocampus).

## Results

### Self-reported Measures

The mean SR score was 10.12 (SD 4.76), and the internal consistency of the scale was good (Cronbach’s *α* = 0.82). Mean BMI was 23.21 (SD 3.74, range: 15.67–37.18). According to the WHO diagnostic categories for the different BMI ranges: 5.3% were obese, 20.2% were overweight, 68.2% had a healthy weight, and 6.3% presented low weight. Partial correlation between SR and BMI, controlling for age and sex, was non-significant (*p* > 0.10). Moreover, we did not find any quadratic relationship between the two variables (*p* > 0.10).

### Morphometry Results

**Figure 1:**
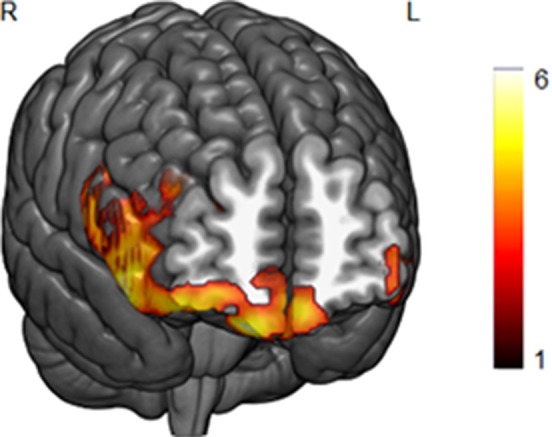
A negative correlation between body mass index (BMI) scores and gray matter (GM) volume in the lateral and medial orbitofrontal cortex in the whole-brain analysis (*p* < 0.05, FWE corrected). Bar color represents *t*-values.

The whole-brain GM analyses with BMI as a single covariate of interest revealed a negative correlation between BMI and GM volume in two prefrontal clusters (*p* < 0.05 FWE cluster-level corrected). The first was a large cluster that extended from the medial orbitofrontal cortex to right lateral prefrontal areas (local maxima at MNI *x*, *y*, *z* = 15, 51, −26, *t* = 5.73, *k* = 8,300), whereas the second was a smaller cluster located in the left middle/inferior prefrontal cortex (local maxima at MNI *x*, *y*, *z* = −48, 48, −9, *t* = 5.07, *k* = 1,313). Both clusters are depicted in Figure 1. We did not obtain any significant positive correlations between BMI and GM volume.

Regarding the analyses with SR scores as a covariate of interest, the whole brain analyses revealed no significant clusters at the pre-established threshold. However, ROI analyses of the possible association between the striatum and the reward sensitivity dimension revealed a negative correlation with the left caudate (*r* = −0.17; *p* < 0.009, FWE corrected).

**Figure 2:**
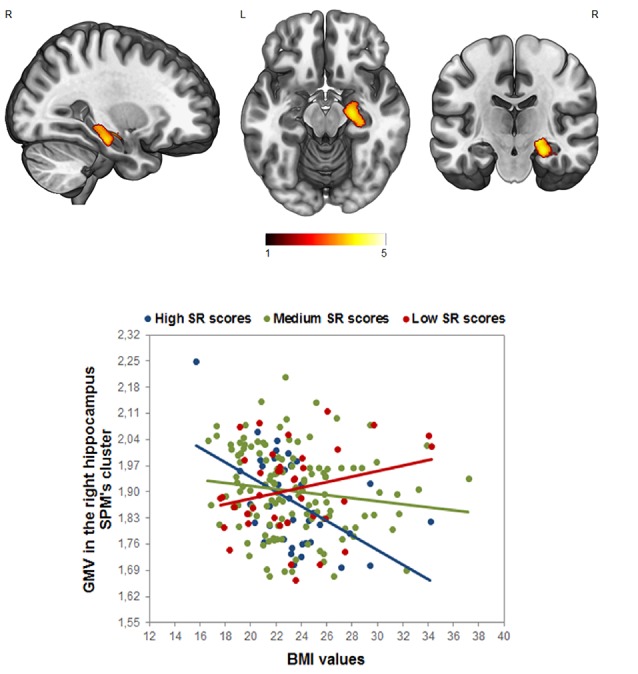
Analyses of the interaction effect between reward sensitivity and BMI in the whole-brain analysis (*p* < 0.05, FWE corrected). Bar color represents *t*-values. (Up) Cluster location in the right hippocampus; (Down) the scatterplot and *R*^2^ is displayed only for visualization purposes. The sample was divided into three groups of high (>1 SD), medium (±1 SD), and low (<1 SD) Sensitivity to Reward (SR), to visualize the GM modulation based on the SR × BMI interaction in the right hippocampus.

Concerning the interaction between the BMI and SR, the whole-brain analysis yielded a significant negative effect between the interaction term (BMI × SR) and the GM volume in the right hippocampus/amygdala (*p* < 0.05 FWE cluster-level corrected, local maxima at MNI *x*, *y*, *z* = 26, −21, −18, *t* = 4.24, *k* = 827; Figure 2). To better visualize this significant interaction, we divided the sample into three groups of high (>1 SD), medium (±1 SD), and low (<1 SD) SR, and we plotted the relationship between BMI and the GM in the right hippocampus. This analysis revealed that participants with high SR scores and high BMI showed a GM volume decrease in this area (see Figure 2). Interestingly, this pattern was also obtained in the left hippocampus at the ROI level (*r* = −0.17, *p* < 0.008 FWE corrected).

## Discussion

Increased BMI is a risk factor for brain atrophy in different areas of the brain, with special emphasis on prefrontal and limbic areas. Moreover, impulsivity and reward sensitivity has been related to reduced GM volume in the striatum and medial and lateral prefrontal cortex. No evidence has been provided, however, about the possible interaction between the two factors. In the present study, we used voxel-wise brain morphometry analysis to investigate the interaction effect of personality and BMI on the brain anatomy in undergraduates. In agreement with previous theoretical approaches, we confirmed that BMI was associated with decreased GM in the bilateral orbitofrontal cortex and that SR scores correlated with lower volume in the striatum. Importantly, we found that the interaction between reward sensitivity and BMI was associated with individual differences in the hippocampal volume. This interaction is mainly driven by the fact that a high BMI in participants with higher SR scores was related to reduced hippocampus volume. To a lesser extent, the interaction also reflects that participants with a high BMI and lower SR scores tended to show increased hippocampus volume. This new finding indicates that high reward sensitivity in overweight individuals may link them to the “outward spiral” of obesity (Hargrave et al., 2016).

Contrary to previous reports (Franken and Muris, 2005; De Decker et al., 2016; van den Berg et al., 2011), the results did not show any kind of relationship between reward sensitivity and BMI. Differences in the questionnaires employed to measure reward sensitivity or differences in the samples (children, adolescents, young adults or adults) may partially explain the discrepancy between studies. However, it is also relevant to remember that RST only predisposes the individual to risky behaviors that may lead to obesity (De Decker et al., 2016; Loxton and Tipman, 2017), but other factors, including metabolic differences, may determine the magnitude of this relationship. Importantly, the present manuscript investigates the interaction between BMI and reward sensitivity, which specifically involves determining the characteristics of the brains of individuals in whom BMI is accompanied by higher scores on reward sensitivity.

As previously found, BMI was directly associated with decreased GM in the right orbitofrontal cortex (Medic et al., 2016; Vainik et al., 2018). This result supports the right brain hypothesis of obesity, which proposes that “hypoactivation in the right prefrontal cortex of obese individuals is related to poor cognitive control of food intake” (Alonso-Alonso and Pascual-Leone, 2007). The reward areas of the brain would be responsible for establishing approach responses to food that should be modulated by the prefrontal cortex. Reduced gray matter in the prefrontal cortex would dysregulate this cognitive control.

Consistent with a wide variety of previous studies, individual differences in sensitivity to different kinds of reward were associated with a reduced striatum volume, especially in the caudate nucleus, as well as in the medial and lateral prefrontal cortex. This result was consistent with previous studies showing that smaller striatal areas were associated with higher scores on reward sensitivity measures (Barrós-Loscertales et al., 2006a; Adrián-Ventura et al., 2019). Diverse psychobiological approaches to personality associate this trait with a different ability to detect rewarding cues and a different probability of making approach responses to these cues (Cloninger et al., 1993; Depue and Collins, 1999). These cues involve different kinds of rewards, including sexual stimuli, monetary incentives, or social recognition (Torrubia et al., 2001). In this regard, recent evidence obtained in healthy populations related a lower volume in striatal nuclei to a preference for approaching specific rewards, including Facebook usage (Montag et al., 2017), pornography consumption (Kühn and Gallinat, 2014), reward delay (Tschernegg et al., 2015), or substance abuse (Urošević et al., 2015). In the same vein, similar reductions in striatal volumes have been found in several pathologies, such as substance abuse disorders (Barrós-Loscertales et al., 2011; Grodin and Momenan, 2017) or ADHD (Shaw et al., 2014). As recently demonstrated for sensitivity to the rewarding properties of music, striatum volume is only a predisposing factor to different kinds of rewards that should be accompanied in the case of music by a specific predisposing factor (Hernández et al., 2019). Here, the interaction with BMI determines the brain associates of high BMI participants whose overweight is associated with stronger reward sensitivity.

The present study shows that the interaction between BMI and reward sensitivity is associated with differences in the volume of the right hippocampus. The interaction is mainly driven by the fact that the combination of high BMI and high SR was related to lower hippocampal volume. The role of the hippocampus in feeding behavior has been established at different levels. At the neuroendocrine level, the hippocampus contains key receptors that signal the energy status of the body and, consequently, modulate the foraging behavior (Kanoski and Grill, 2017). This converts the hippocampus into the brain area that integrates interoceptive information obtained from endocrine markers with external information. Recent research shows that in hunger states, the functional connectivity between the striatum and the hippocampus is potentiated (Contreras-Rodriguez et al., 2019), and that the stronger connectivity between the two structures is associated with impulsivity and BMI (Sharkey et al., 2019).

At the personality level, the RST model relates the septohippocampal system to an activation of memories that may serve to resolve conflicts (Gray and McNaughton, 2000). Recent evidence shows that the hippocampus contributes to behavioral avoidance and choice monitoring during approach-avoidance conflicts (Loh et al., 2017). During these conflicts, stronger reward sensitivity would activate the striatum more, promoting goal-directed approach responses to obtain rewards. This greater activation and motivation to obtain rewards would facilitate the emission of previously rewarded responses and diminish the probability of the responses being modulated by memory-guided previous negative experiences that would depend on the hippocampus (Patterson and Newman, 1993).

Although less relevant, a second pathway to obesity is observed in individuals with a predisposition to negative emotions, that is, those with lower scores on reward sensitivity and increased hippocampus volume. Previous studies have demonstrated that low reward sensitivity predisposes people to depression (Pinto-Meza et al., 2006). Furthermore, increased hippocampus volume has been observed in individuals with elevated trait anxiety (Barrós-Loscertales et al., 2006b; Cherbuin et al., 2008). We speculate that both factors would contribute to some kind of emotional eating that would lead to overweight.

Given its crucial role in regulating feeding behavior, it is relevant to define the consequences of lower hippocampal volume. In this regard, Hargrave et al. (2016) proposed the concept of the outward spiral to illustrate how the hippocampus becomes damaged in different situations, including obesogenic environments, exposure to environmental toxins, early exposure to western diets, or congenital hippocampal deficits. These conditions would facilitate the beginning of a vicious cycle where reduced hippocampal volume reduces the capacity of satiety signals to inhibit approach behavior toward high-calorie foods that lead to weight gain, which then harms the hippocampus. Thus, high BMI and stronger reward sensitivity are directly related to hippocampal damage.

## Conclusions

This study demonstrates the interactive effect of the reward sensitivity and the BMI on the GM volume of the hippocampus. Previous studies have shown that BMI was associated with reduced brain volume in the orbitofrontal cortex and that lower volume in the striatum was typically observed in individuals with stronger reward sensitivity. Both results have been replicated in our study. Importantly, we have also shown that the interaction between high reward sensitivity and a high BMI was associated with reduced hippocampal volume. This result would indicate a dysfunctional hippocampus, which would reduce the probability of inhibiting reward-directed feeding behavior during approach-avoidance conflicts. In sum, this study contributes to demonstrating that stronger reward sensitivity may predispose people to obesity.

## Data Availability Statement

The data that support the findings of this study are available from the corresponding author upon reasonable request.

## Ethics Statement

The studies involving human participants were reviewed and approved by Univeristat Jaume I. The patients/participants provided their written informed consent to participate in this study.

## Author Contributions

The study was designed by MP and CÁA. Experiments were conducted by MP, JA-V, and VC and analyzed by JA-V and VC. MP and CÁA wrote the article.

## Conflict of Interest

The authors declare that the research was conducted in the absence of any commercial or financial relationships that could be construed as a potential conflict of interest.

## Footnotes

^1^http://dbm.neuro.uni-jena.de/vbm/

^2^http://Neuromorphometrics.com/

^3^http://www0.cs.ucl.ac.uk/staff/g.ridgway/vbm/get_totals.m
